# 

**Published:** 1877-02

**Authors:** 


					﻿Vol. 24.	FEBRUARY, 1877.	No. 2.
TWENTY-FOURTH YEAR.
HALL’S
Journal of Health.
PUBLISHED AT 137 EIGHTH STREET, NEW YORK,
Four Doors East of 750 Broadway.
E. H. GIBBS, M.D., Editor.
AGENTS FOR THE TRADE;
AMERICAN NEWS COMPANY, 121 NASSAU STREET.
NEW YORK NEWS COMPANY, 18 BEEKMAN STREET.
' Terms, 91.50 a Year.
SINGLE NUMBER, 15 CENTS.
Jm Kbnt, Printer, 11 Frankfort St., New Tork.
				

